# Validity assessment of quantitative light-induced fluorescence-digital (QLF-D) for the dental plaque scoring system: a cross-sectional study

**DOI:** 10.1186/s12903-018-0654-8

**Published:** 2018-11-20

**Authors:** Jong-Bin Lee, Da-Hye Choi, Yon-Joo Mah, Eun-Kyoung Pang

**Affiliations:** 1 Department of Periodontology, Mokdong Hospital, Ewha Womans University, Seoul, South Korea; 2 Department of Clinical Oral Health Science, Graduate School of Clinical Dentistry, Ewha Womans University, Seoul, South Korea; 3 Department of Pediatric Dentistry, Mokdong Hospital, Ewha Womans University, Seoul, South Korea; 4 Department of Periodontology, College of Medicine, Ewha Womans University, 1071 Anyangcheon-ro, Yangcheon-gu, Seoul, 07985 Republic of Korea

**Keywords:** Dental plaque, Quantitative light-induced fluorescence-digital, Gingival index, Bleeding on probing, Probing pocket depth, Patient hygiene performance index

## Abstract

**Background:**

The aim of this study was to analyze the correlation between the dental plaque indices measured using quantitative light-induced fluorescence-digital (QLF-D) and conventional clinical indices that assess gingival status.

**Methods:**

From among the patients who visited Ewha Womans University Mokdong Hospital, 33 adults in their 20s who had relatively even teeth were selected for full-mouth QLF-D imaging. The images were used to analyze the QLF-D score and the QLF-D ΔR score. As clinical indices, the gingival index (GI), bleeding on probing (BOP), probing pocket depth (PPD), and patient hygiene performance (PHP) index were measured. The correlations between the QLF-D score and QLF-D ΔR score and each clinical index were analyzed. Analyses were performed comparing the indices of maxillary and mandibular teeth, the teeth on right and left sides of the mouth, anterior and posterior teeth, and buccal and lingual surfaces of each tooth. Pearson’s correlation analysis was conducted (*p* < 0.05).

**Results:**

The mean full-mouth QLF-D score was highly correlated with the GI, BOP, PPD, PHP index (*p* < 0.01). The mean full-mouth QLF-D score showed the highest correlation with GI (*r* = 0.749) and the lowest correlation with PPD (*r* = 0.683). The correlations between the QLF-D score were higher in the mandible than in the maxilla and in the anterior teeth than in the posterior teeth, while no significant differences were seen between the buccal and lingual surfaces of tooth.

**Conclusions:**

This study concluded that the correlations between the plaque indices measured for each tooth surface area using QLF-D and the clinical indices assessed were significantly high, and it allowed objective determination of the gingival status. Therefore, the plaque index measured using QLF-D may be used as an alternative to supplement the shortcomings of conventional clinical indices for educating patients about plaque control and continued patient oral care.

**Electronic supplementary material:**

The online version of this article (10.1186/s12903-018-0654-8) contains supplementary material, which is available to authorized users.

## Background

Irritant extraneous substances that stick to the tooth surface include bacterial plaques, dental calculi, residual proteins from food, and colored substances, among which dental plaques and dental calculi are the most important localized causes of periodontal diseases [1]. Oral biofilm, one of the main causes of periodontal diseases, is formed mainly by bacteria, and is clinically important [2] because it is a key pathogenic predecessor substance for dental calculi.

The correlation between oral biofilm and gingivitis was first established by Loe et al. [3] in 1965. According to them, chronic stimuli can be applied to the gingiva to cause periodontal diseases unless oral biofilms are periodically removed. Thus, the removal of oral biofilms is one of the most basic and effective processes for preventing oral cavity diseases [4].

Basic education regarding oral biofilms is necessary for preventing oral diseases by keeping the oral environment clean. In this basic education, clinical specialists educate patients regarding oral biofilms in an easily understandable format to help them develop effective oral biofilm removal methods tailored to their needs by their own maintenance, which can help the patients with a life-long habit of keeping their oral environment clean.

A method to quantify the oral biofilms is necessary for determining the oral health of patients periodically. Presently, both in clinical practice and in periodontal disease studies, various indices for oral biofilm observation for periodontal disease determination (e.g., gingivitis and periodontitis) are introduced. These indices were created to allow easy comparison between groups for quantifying oral clinical conditions using the same criteria and methods. To be used clinically and in studies, these dynamic indices must be easily usable by patients, should simultaneously test many patients in a short period of time, indicate the clinical conditions objectively, be highly reproducible, be fit for statistical analysis, and be closely linked to the clinical conditions of specific diseases [5].

Clinical measurement indices, which are mainly used at present for oral condition observation in determining periodontal disease in clinical practice, include gingival indices for evaluating the health conditions of the gingiva at each stage, such as gingival index (GI), bleeding on probing (BOP), probing pocket depth (PPD), and patient hygiene performance (PHP) index that can quantitatively evaluate the degrees of dental plaque adhesion.

The most commonly used method for measuring dental plaque accumulation in clinical practice is to stain the attached dental plaques with dyes [6, 7]. Among these dyeing methods for evaluating patient hygiene performance, a method of staining the oral biofilms on a each tooth surface divided into five parts and recording the presence of dental plaques is known to be useful for educational purposes, and as the most effective method for calculating the quantified ratios of oral biofilm areas [8]. However, these existing methods are performed by direct visual observation of the patient’s oral condition, and thus lead to evaluator bias and judgment errors. Furthermore, the method has limitations in that much time and cost are required for educating the evaluators on precise measurements of the oral conditions [9].

An early quantitative light-induced fluorescence (QLF) method developed recently has been introduced in the form of an oral camera equipment loaded with software that can quantitatively analyze the fluorescence loss in the area of erosion using the autofluorescence of the teeth induced by blue visible light at a wavelength of 405 nm, which detects the mineral changes occurring in the teeth [10]. QLF can quantitatively detect the subtle changes in the minerals within the teeth during incipient caries in detail, and is being used in the field for evaluating and studying the occurrence and progress of incipient caries [11].

QLF-digital (QLF-D), can be used for evaluating and studying incipient caries as well as for measuring dental plaque areas by simple photography and quantifying dental plaques. Furthermore, QLF-D can detect dental plaques in red fluorescence that appear due to porphyrin produced by oral bacteria, which allows for an objective detection of even small amounts of dental plaque changes [12]. Unlike other dental plaque measurement methods that stain the dental plaques using dyes and distinguish the differences of images using computer software (e.g., Adobe Photoshop), QLF-D does not require coloring of the dental plaques adhered to the tooth surface for measurement, and can be applied easily, thus avoiding patient complaints regarding difficulty in removing the stain from their tongue or lips after dye usage [13]. Moreover, QLF-D can be used for the purpose of customized oral hygiene maintenance education based on an individual’s hygiene performance because it allows quantification of oral biofilm maturation.

Previous studies that measured the degrees of dental plaque adhesion using QLF-D include a study in 2013 that investigated if QLF can be used in an oral hygiene process [11], a study on various types of oral rinsing solutions and their oral biofilm decreasing effects using QLF-D [14], another study [9] on developing new dental plaque measurement methods using QLF-D, and recently, a study [15] to evaluate the clinical application potential of oral biofilm test methods using QLF-D.

Based on the previous study results on dental plaque measurement methods using QLF-D, Han et al. [9] confirmed that QLF-D can be used as a dental plaque measurement tool for measuring old dental plaques that have strong clinical relation to diseases of the oral cavity. Hwang et al. [15] reported the full potential of QLF-D as a new dental plaque measurement method by validating that it can be used in monitoring oral biofilm and gingival conditions. However, there are limitations in applying QLF-D for evaluating the overall oral conditions based on the results of these existing studies since they targeted only the upper and lower mandibular anterior teeth region because of the difficulty in measuring the maxillary posterior teeth area, palatal and lingual surfaces of tooth due to the constraints in photographing these areas.

Thus, in this study, all oral biofilm indices were measured for full mouth areas using QLF-D, and the correlation between GI, BOP, PPD, PHP index was analyzed followed by a comparative analysis between each part to evaluate if an oral biofilm measurement method using QLF-D can be used as an alternative for existing methods for full mouth areas regardless of the different regions.

## Methods

### Materials

This study was conducted with the approval of Institutional Review Board of the College of Medicine, Ewha Womans University (Approval no. 15–11-01). The study was performed with 33 adult patients aged over 20, recruited from among the outpatients who visited the Ewha Womans University Mokdong Hospital, agreed to the terms of the experiment (Additional file 1), had a sound tooth surface in the upper and lower mandibular anterior teeth region and posterior teeth area, and had relatively even teeth arrangement.

The number of subjects required for this study was calculated to be 33 using G Power 3.1.9, coefficient 0.3, error rate 20%, and statistical power of 80%. Among patients, those with orthodontic bracket placed, severe crowding of teeth not visually verified in the photograph, fixed or implant restoration installed, less than 24 teeth, and serious systemic diseases were excluded from the study.

QLF-D score was measured using QLF-D (Biluminator; Inspector Research Systems BV, Amsterdam, Netherland) to evaluate the quality and quantity of the adhered oral biofilm, and the QLF-D specifications are presented in Table 1.

**Table 1:** Photographing condition of QLF-D in this study

	White light	Blue light
Shutter speed	1/60 s	1/30 s
Aperture value	8.0	5.6
White balance	manual	daylight
ISO speed	ISO1600	ISO1600
Pixel size	2592 × 1728	2592 × 1728

### Methods

The participants visited the hospital for testing in the afternoon without brushing their teeth after lunch. A self-reporting questionnaire was used to survey the general characteristics and oral care methods used by the participants, and various factors from the questionnaire, such as sex, age, occupation, existence of systemic disease, use of toothbrush and other oral hygiene products, periodic scaling, and smoking status, were investigated (Additional file 2).

**Fig. 1:**
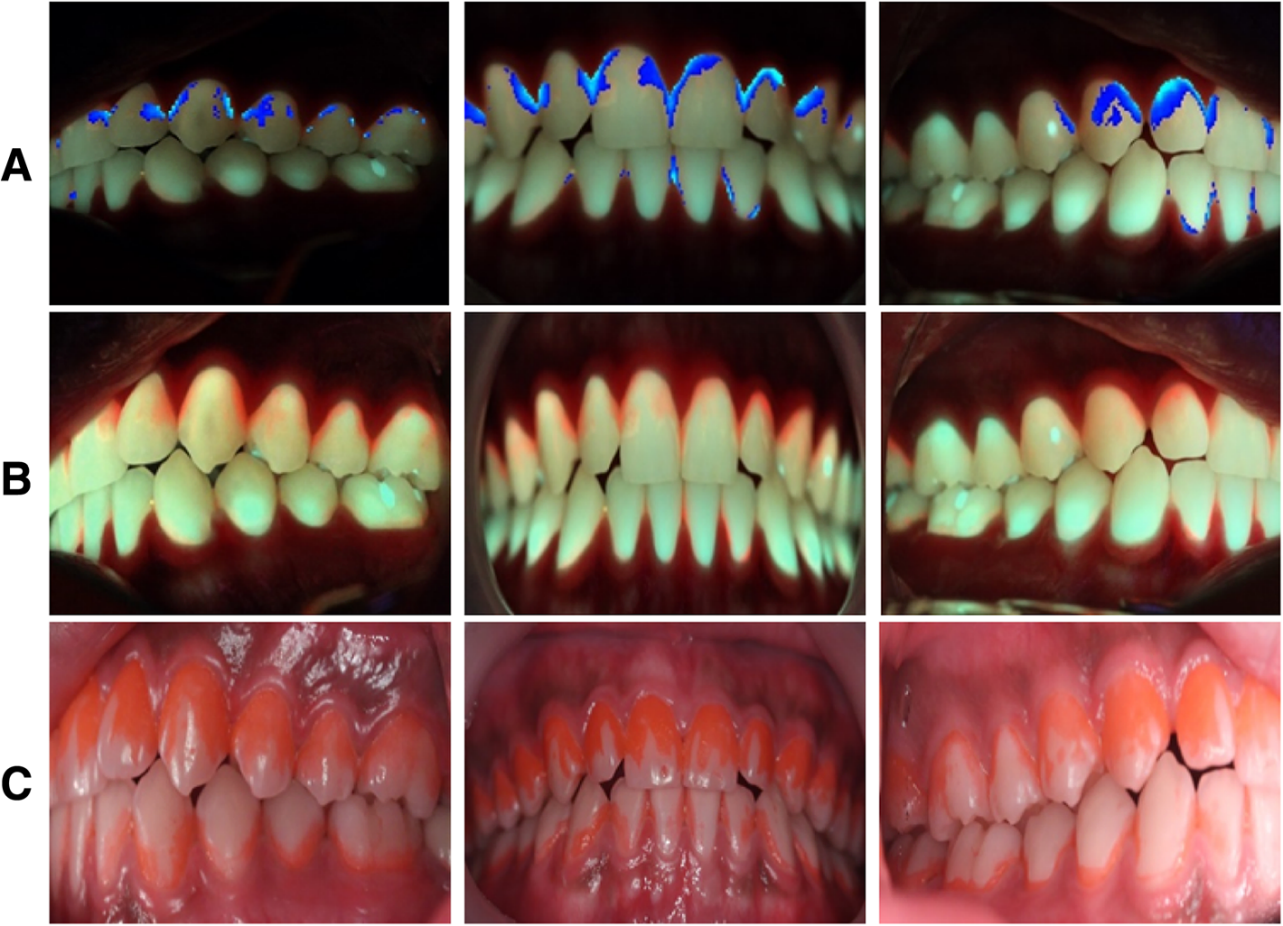
Photographs of plaque in the buccal (labial) side of teeth by QLF-D scores. **a** Plaque areas analyzed by software. **b** Under the blue light. **c** Application disclosing solution

**Fig. 2:**
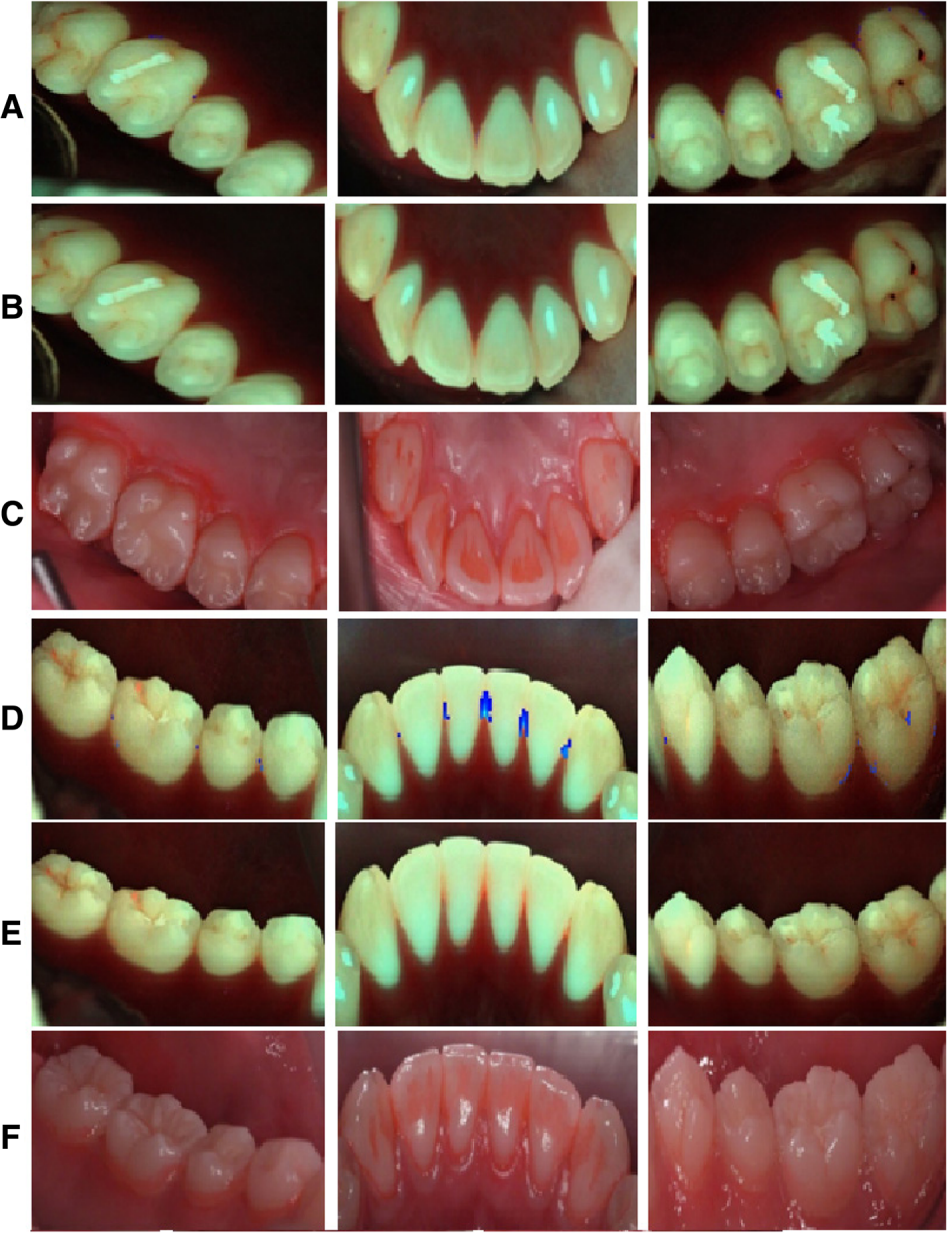
Photographs of plaque in the lingual (palatal) side of teeth by QLF-D scores. **a**, **b**, **c** Maxilla. **d**, **e**, **f** Mandible. **a**, **d** Plaque areas analyzed by software. **b**, **e** Under the blue light. **c**, **f** Application of disclosing solution

The test area was divided into a total of 8 areas, including the maxilla and the mandible, left and right sides, anterior and posterior teeth, and buccal and lingual surfaces of teeth. QLF-D images of these areas were acquired using EOS 650D (Cannon, Tokyo, Japan) (Figs. 1 and 2). The images from QLF-D were used to analyze the QLF-D score and ΔR values in different parts of the mouth using a QLF-D analysis program (QA2 v 1.23; Inspektor Research Systems BV, Amsterdam, Netherlands). To determine the gingival status of the participants, the GI, BOP and PPD were measured for each tooth, after which the PHP index was measured by staining the plaque with disclosing solution.

For elimination of inter-evaluator errors, a single dental hygienist, with over 5 years of clinical experience, measured the QLF-D score and clinical measurement indices of all participants. The results of all measurement were recorded in Additional file 3.

### QLF-D score

The Simple Plaque Score (SPS) was used for quantitative and qualitative assessment of dental plaque deposits, and scores ranging from 0 to 5 points were assigned according to the attached area of dental plaque using QA2 v1.23, a QLF-D analysis program.

### QLF-D ΔR score

A strong red fluorescence can be seen with a greater degree of maturation of dental plaque. In this study, the dental plaque was assessed with sub-scores of ΔR30, ΔR70, and ΔR120 according to the fluorescence intensity. Higher ΔR values indicate areas with more active bacterial metabolism within the dental plaque, representing a greater level of dental plaque maturation [15].

### Gingival index (GI)

The GI described by Löe and Silness was used to measure the gingivitis status. Gingivitis was assessed by dividing each tooth surface into the buccal, lingual, mesial, and distal surfaces and using a periodontal probe to evaluate the health status of the gingival tissues. The force applied during probing was controlled, and the pocket depth or bone loss was not measured. The measurement results were scored with 0 to 3 points, and the mean measured value from each tooth was used as the representative value. The classification criteria are shown below:

- 0 point = (no inflammation) normal gingiva.
- 1 point = (mild gingivitis) slight color change and no swelling or BOP.
- 2 points = (moderate gingivitis) accompanied by redness, swelling, and/or BOP.
- 3 points = (severe gingivitis) distinct redness and swelling; natural gingival bleeding and ulceration.

### Bleeding on probing (BOP)

A periodontal probe (Marquis probe, Hu-Friedy, USA) was inserted into the mesial and distal surfaces of each tooth in the apical direction up to the junctional epithelium with a pressure below 20 g. After 20 s, a determination was made regarding whether there was gingival bleeding. Based on the determination of bleeding from the mesial and distal surfaces of each tooth, 1 point was given for cases with bleeding and 0 points for cases with no bleeding. Then, the mean score for each tooth was derived and used as the representative value [16].

### Probing pocket depth (PPD)

A periodontal probe (Marquis probe, Hu-Friedy, USA) was used to measure the distance between the gingival margin and the base of periodontal pocket or gingival sulcus. The distance was determined by measuring 6 areas, including the mesio-buccal surface, the center of the buccal surface, the disto-buccal surface, the mesio-lingual surface, the center of the lingual surface, and the disto-lingual surface. Then, the mean of the measured values for each tooth was used as the representative value.

### Patient hygiene performance (PHP) index

Podshadley Haley’s PHP index was used to assess each patient’s degree of dental plaque deposition and patient hygiene performance. Examinations were performed on a total of 5 areas by dividing each tooth surface into 3 parts corresponding to the mesial, central, and distal areas and then further dividing the central area into the gingival, central, and occlusal surface. After staining the plaque with plaque dye (IC-Disclosing solution, Il-chung dental co., Seoul, Korea), each area was scored as 1 point if the colored area persisted and as 0 points if it did not. In other words, if all 5 areas on a single tooth were stained, the score would be 5 points, whereas if none of the areas were stained, then the score would be 0 points. The mean value for each tooth was designated as the representative value, and the sum of all represent values was divided by the number of measured teeth to derive the mean.

### Statistical analysis

Statistical analyses were performed using IBM SPSS Statistics 21.0 (IBM Co., Armonk, NY, USA). To identify the correlations of the QLF-D score and the QLF-D score ΔR value of each tooth with the clinical measurement indices, Pearson’s correlation analysis was performed (*p* < 0.05).

## Results

The general characteristics of the study participants were presented in Table 2.

**Table 2:** General characteristics of the subject

Variable	Group	N	Percentage (%)
Gender	Male	14	42.4
	Female	19	57.6
Age	20~ 29	23	67.6
	30~ 39	9	26.5
	40~ 49	0	0
	50~ 59	1	2.9
Scaling	Yes	9	27.3
	No	24	72.7
Smoking	Yes	6	18.2
	No	27	81.8
Oral hygiene product	Use	12	36.4
	No use	21	63.6
Total		33	100

### Correlation analysis between full-mouth QLF-D scores and clinical indices

The mean full-mouth QLF-D score showed significantly high correlations with the GI, BOP, PPD, and PHP index (*p* < 0.01). Except of the correlation with the ΔR value, the mean full-mouth QLF-D score showed the highest correlation with the GI among all of the clinical indices analyzed (*r* = 0.749), and it showed the lowest correlation with PPD (*r* = 0.683).

The QLF-D score ΔR values showed high correlations with all clinical indices, and higher ΔR values were less well correlated with the clinical measurement indices (*p* < 0.05) (Table 3).

**Table 3:** Correlation coefficients of among the QLF-D scores, PHP score, GI scores, BOP score, and PD scores (*N* = 33)

	QLF-D score	Δ R 30	Δ R 70	Δ R 120	PHP index	GI	BOP	PD
QLF-D score	1							
Δ R 30	0.966^**^	1						
Δ R 70	0.816^**^	0.810^**^	1					
Δ R 120	0.628^**^	0.621^**^	0.924^**^	1				
PHP index	0.730^**^	0.760^**^	0.598^**^	0.466^**^	1			
GI	0.749^**^	0.785^**^	0.553^**^	0.403^*^	0.805^**^	1		
BOP	0.730^**^	0.761^**^	0.536^**^	0.368^*^	0.801^**^	0.955^**^	1	
PD	0.683^**^	0.708^**^	0.613^**^	0.496^**^	0.598^**^	0.782^**^	0.738^**^	1

*QLF-D* Quantitative Light-Induced Fluorescence-Digital, *QLF-D ⊿R score* redness differences of 30, 70, and 120% between the teeth and the red plaque observed on the QLF-D photograph, *PHP index* Patient hygiene performance index, *GI* Gingival index, *BOP* Bleeding on probing, *PPD* Probing pocket depth

^*^P < 0.05

^**^P < 0.01

### Correlation analysis between the QLF-D scores and the clinical indices in the maxilla and the mandible

The mean QLF-D scores were highly correlated with the mean values of all clinical indices in the maxilla and the mandible (*p* < 0.05). In the maxilla, the QLF-D score showed the highest correlation with the PHP index (*r* = 0.737) and a relatively low correlation with PPD (*r* = 0.565) (*p* < 0.01) (Table 4). The QLF-D score also showed the highest correlation with the PHP index (*r* = 0.794), and it showed the lowest correlation with PPD (*r* = 0.583) in the mandible (Table 4).

**Table 4:** Correlation coefficients of labial (buccal)/palatal (lingual) side of teeth among the QLF-D scores, PHP score, GI scores, BOP score and PD scores (*N* = 33)

	QLF-D score	Δ R30	Δ R70	Δ R120	PHP	GI	BOP	PD
Labial (buccal)								
QLF-D score	1							
Δ R30	0.971^**^	1						
Δ R70	0.684^**^	0.670^**^	1					
Δ R120	0.290	0.259	0.836^**^	1				
PHP	0.790^**^	0.772^**^	0.471^**^	0.100	1			
GI	0.776^**^	0.787^**^	0.414^*^	0.015	0.770^**^	1		
BOP	0.756^**^	0.768^**^	0.392^*^	−0.015	0.807^**^	0.940^**^	1	
PD	0.621^**^	0.632^**^	0.339	0.031	0.570^**^	0.768^**^	0.757^**^	1
Lingual (palatal)								
QLF-D score	1							
Δ R30	0.946^**^	1						
Δ R70	0.884^**^	0.930^**^	1					
Δ R120	0.772^**^	0.847^**^	0.964^**^	1				
PHP	0.723^**^	0.746^**^	0.706^**^	0.660^**^	1			
GI	0.702^**^	0.682^**^	0.613^**^	0.525^**^	0.794^**^	1		
BOP	0.685^**^	0.648^**^	0.608^**^	0.517^**^	0.706^**^	0.906^**^	1	
PD	0.698^**^	0.703^**^	0.679^**^	0.597^**^	0.617^**^	0.678^**^	0.645^**^	1

*QLF-D* Quantitative Light-Induced Fluorescence-Digital, *QLF-D ⊿R score* redness differences of 30, 70, and 120% between the teeth and the red plaque observed on the QLF-D photograph, *PHP index* Patient hygiene performance index, *GI* Gingival index, *BOP* Bleeding on probing, *PPD* Probing pocket depth

^*^P < 0.05

^**^P < 0.01

Analysis of the correlation between the maxillary and mandibular QLF-D scores and clinical indices demonstrated that the mandible tended to show relatively higher correlations than the maxilla for all clinical indices.

### Correlation analysis between the QLF-D scores and the clinical indices in the anterior and posterior teeth

The QLF-D scores of the anterior and posterior teeth demonstrated significantly high correlations with the clinical indices in all cases, with the exception of the correlation between the QLF-D score ΔR 120 value of the posterior teeth and the GI and the correlations between the QLF-D score ΔR 70 and ΔR 120 values of the posterior teeth and the BOP (*p* < 0.05).

The QLF-D score of the anterior teeth showed the highest correlation with the PHP index among all clinical indices (*r* = 0.800), and it showed a relatively low correlation with the PPD (*r* = 0.410) (Table 5).On the other hand, the QLF-D score of posterior teeth showed the highest correlation with PPD (*r* = 0.694) and a relatively low correlation with BOP (*r* = 0.541) (Table 5).

**Table 5:** Correlation coefficients of maxilla/mandible among the QLF-D scores, PHP score, GI scores, BOP score and PD scores (*N* = 33)

	QLF-D score	Δ R30	Δ R70	Δ R120	PHP	GI	BOP	PD
Maxilla								
QLF-D score	1							
Δ R30	0.954^**^	1						
Δ R70	0.644^**^	0.629^**^	1					
Δ R120	0.350^*^	0.302	0.878^**^	1				
PHP	0.737^**^	0.745^**^	0.373^*^	0.136	1			
GI	0.647^**^	0.685^**^	0.228	−0.022	0.723^**^	1		
BOP	0.614^**^	0.677^**^	0.227	−0.042	0.725^**^	0.946^**^	1	
PD	0.565^**^	0.516^**^	0.252	0.071	0.440^*^	0.695^**^	0.637^**^	1
Mandible								
QLF-D score	1							
Δ R30	0.931^**^	1						
Δ R70	0.838^**^	0.904^**^	1					
Δ R120	0.703^**^	0.772^**^	0.948^**^	1				
PHP	0.794^**^	0.751^**^	0.679^**^	0.601^**^	1			
GI	0.771^**^	0.762^**^	0.664^**^	0.560^**^	0.834^**^	1		
BOP	0.779^**^	0.766^**^	0.700^**^	0.610^**^	0.830^**^	0.948^**^	1	
PD	0.583^**^	0.650^**^	0.621^**^	0.567^**^	0.610^**^	0.731^**^	0.726^**^	1

*QLF-D* Quantitative Light-Induced Fluorescence-Digital, *QLF-D ⊿R score* redness differences of 30, 70, and 120% between the teeth and the red plaque observed on the QLF-D photograph, *PHP index* Patient hygiene performance index, GI: Gingival index, *BOP* Bleeding on probing, *PPD* Probing pocket depth

^*^P < 0.05

^**^P < 0.01

The mean QLF-D scores of the anterior teeth were more strongly correlated with the clinical indices than those of the posterior teeth.

### Correlation analysis between the QLF-D scores and the clinical indices in the buccal and lingual tooth surface

The QLF-D score of the buccal surface demonstrated the significantly high correlations with all clinical indices, with the exception of the correlations between the QLF-D score ΔR 70 value and PPD and between the QLF-D score ΔR 120 value and the clinical indices (*p* < 0.05). The QLF-D score of the buccal surface showed the highest correlation with the PHP index (*r* = 0.790) and the lowest correlation with PPD (*r* = 0.621) (Table 6).

**Table 6:** Correlation coefficients of anterior/posterior teeth among the QLF-D scores, PHP score, GI scores, BOP score and PD scores (*N* = 33)

	QLF-D score	Δ R30	Δ R70	Δ R120	PHP	GI	BOP	PD
Anterior teeth								
QLF-D score	1							
Δ R30	0.922^**^	1						
Δ R70	0.818^**^	0.822^**^	1					
Δ R120	0.444^**^	0.439^*^	0.820^**^	1				
PHP	0.800^**^	0.780^**^	0.756^**^	0.489^**^	1			
GI	0.757^**^	0.752^**^	0.576^**^	0.340	0.778^**^	1		
BOP	0.748^**^	0.761^**^	0.674^**^	0.462^**^	0.750^**^	0.868^**^	1	
PD	0.410^*^	0.515^**^	0.548^**^	0.477^**^	0.518^**^	0.535^**^	0.535^**^	1
Posterior teeth								
QLF-D score	1							
Δ R30	0.976^**^	1						
Δ R70	0.823^**^	0.826^**^	1					
Δ R120	0.700^**^	0.709^**^	0.952^**^	1				
PHP	0.682^**^	0.717^**^	0.499^**^	0.421^*^	1			
GI	0.674^**^	0.680^**^	0.469^**^	0.357^*^	0.756^**^	1		
BOP	0.541^**^	0.555^**^	0.302	0.202	0.696^**^	0.842^**^	1	
PD	0.694^**^	0.692^**^	0.541^**^	0.455^**^	0.557^**^	0.806^**^	0.661^**^	1

*QLF-D* Quantitative Light-Induced Fluorescence-Digital, *QLF-D ⊿R score* redness differences of 30, 70, and 120% between the teeth and the red plaque observed on the QLF-D photograph, *PHP index* Patient hygiene performance index, *GI* Gingival index, *BOP* Bleeding on probing, *PPD* Probing pocket depth

^*^P < 0.05

^**^P < 0.01

The QLF-D score for the lingual surface showed significant correlations with all clinical indices (*p* < 0.05). The highest correlation was with the PHP index (*r* = 0.723), and the lowest correlation was with the BOP (*r* = 0.685) (Table 6).

The QLF-D scores from the buccal and lingual teeth surfaces showed mostly similar correlations with the clinical measurement indices.

## Discussion

Conventional methods of expressing dental plaque include O’Leary Index (plaque control record, PCR) score, plaque index, and tooth coloring [17, 18]. These direct and visual observation methods which dyed or directly measured teeth and periodontal tissue are inconvenient, inaccurate, and waste of time. Also, they may be limited in detection due to the presence of saliva and the mixture of dye and plaque [19]. However, if QLF-D is used, the time required for dental hygiene process can be saved, and the convenience, accuracy, and efficacy of dental hygiene can be improved. For example, it is easy to confirm whether the dentin bacteria layer has been completely removed before the sealant procedure [11].

To overcome the disadvantages of conventional plaque indices, the planimetric method was introduced [20]. This method is a method of calculating or analyzing area on the photographic images after teeth dyeing with a hand tracing or a computer digitizing. Computer digitizing is more accurate and objective than conventional methods. However, this method has the disadvantage that the plaque area is analyzed, but the plaque depth is not measured. In contrast, QLF-D is capable of measuring the overall plaque distribution (i.e., area and depth) and evaluating the plaque maturity based on a fluorescence differentiation. Also, since there are various plaque detection methods, teeth dyeing is not essential, and it is easy to use and portable by using a small camera. It can exclude flashlight, distortion, and specular reflections of images that affect planimetric analysis [20].

The present study used QLF-D to measure the dental plaque index of all teeth and analyzed the correlations with clinical indices that are commonly used in the clinical situation to evaluate the periodontal status, such as the GI, BOP, PPD, and PHP index. The findings were then compared and analyzed for specific areas (maxilla & mandible, anterior teeth & posterior teeth, and buccal & lingual surface of teeth) to assess whether QLF-D can be used as an alternative to existing examination methods to evaluate the overall periodontal status.

This study obtained a correlation coefficient of 0.730 for the correlation between the QLF-D score and the mean full-mouth PHP index and a correlation coefficient of 0.749 for the correlation between the QLF-D score and the mean full-mouth GI. Moreover, the correlation coefficients for the QLF-D scores and the mean score for the full-mouth BOP and the PPD were 0.730 and 0.683, respectively, showing significantly strong positive correlations in all cases (*p* < 0.05). The GI, BOP, and PHP index are clinical indices that directly reflect the state of gingivitis caused by dental plaque; therefore, their correlations appeared to be high. On the other hand, PPD is not directly affected by dental plaque, and as a result, its correlation appeared relatively low.

Pearson’s correlation analysis of the maxillary and mandibular QLF-D results and clinical indices showed that QLF-D had significantly high positive correlations with all of the indices studied (*p* < 0.01). Both the maxillary and mandibular QLF-D scores showed the highest correlation with the PHP index, which can be considered sufficient evidence that the QLF-D score is reliable as a dental plaque-screening tool. Moreover, in all clinical indices, the correlation coefficients appeared higher in the mandible than in the maxilla, which is believed to be the result of easier QLF-D imaging and easier control of this device in the mandible than in the maxilla.

Analyses of the correlations between the QLF-D scores and the clinical indices in the anterior and posterior teeth showed significantly high correlations, with the exception of the QLF-D score ΔR 120 value and GI in the anterior teeth and the QLF-D score ΔR 70 and ΔR 120 values and the BOP in the posterior teeth (*p* < 0.05). It is conceived that the reason why QLF-D score ΔR 120 value showed slightly lower correlations with the clinical indices was because most of the participants were young and had a good periodontal status and lower overall dental plaque maturity.

The analyses of the correlation between the mean QLF-D scores and the clinical indices in the buccal and lingual surfaces showed significantly high correlations, with the exception of the correlation between the buccal QLF-D score ΔR 70 value and PPD and between the QLF-D score ΔR 120 value and clinical indices (*p* < 0.05). This is most likely due to the young age of the participants. Similarly, in the correlation analyses of the anterior teeth and posterior teeth, the maturity of the dental plaque on the buccal and lingual surfaces was too low to show the slightly lower correlations.

In a study by Hwang et al. [15] that assessed the clinical applicability of QLF-D for measuring dental plaque, the QLF-D score of the anterior teeth showed a significant correlation with the GI, with correlation coefficient of 0.562. The QLF-D score of the anterior teeth and the PCR score for the anterior teeth had a correlation coefficient of 0.638, which was similar to the results in the present study (*p* < 0.01).

In this study, the mean full-mouth QLF-D score was highly correlated with the GI, BOP, PPD, and PHP index (*p* < 0.01). Also, the correlations of the QLF-D score were higher in the mandible than in the maxilla and in the anterior than in the posterior, while similar correlations were seen between the buccal and lingual surfaces of teeth. In other words, this study results are similar to the previous study results [15]. However, some of higher QLF-D score ΔR values showed low correlations with all clinical measurement indices (*p* < 0.05). These results are due to the fact that our study subjects are relatively young and have a low maturity level of dental plaque compared to the subjects of previous studies. In a previous study in which the age of study subjects was relatively high, the participants were classified into the healthy gingiva group and gingivitis group according to the conventional classification criteria of GI. QLF-D scores were statistically significantly higher (*p* = 0.007) in the gingivitis group (1.71 ± 1.545) than the healthy gingiva group (0.74 ± 1.290). It is deemed that the red fluorescence from the mature supragingival plaque detected by QLF-D can be useful for monitoring the state of gingivitis. [15] The maturation of dental plaque is important for measurements using QLF-D.

In summary, the correlations between the dental plaque index measured for each tooth surface area using QLF-D and the clinical indices assessed were significantly high. Therefore, QLF-D as a method for dental plaque examination may be an alternative that can compensate for the shortcomings of existing indices based on an assessor’s visual inspection. Furthermore, because QLF-D enables a more objective analysis than conventional methods that may vary according to the assessor’s subjective view, QLF-D can be used for continued patient care. Also, in order to increase the accuracy of abnormal symptoms detection in the oral cavity and to facilitate the convenience of clinical use, QLF-D is used [11]. It is further considered that QLF-D can be used in large-scale examinations to reduce the examination time and cost [21]. A recently developed Q-scan device (AIOBIO, Seoul, Korea) allows nonprofessionals to visualize and detect dental plaque easily, thereby enabling oral hygiene care to be managed in the home [22].

The present study was limited due to the small sample size and the fact that the participants were mostly in their 20s and 30s and hence did not represent all age groups. Therefore, future studies should include a wider range of age groups and a larger participant population.

## Conclusions

In the present study, we analyzed the correlations between the QLF-D scores from all teeth surface areas and clinical indices and the assessment of the clinical applicability of QLF-D. Within the limits of the present study, it is concluded that the correlations between the plaque index measured using QLF-D and the clinical indices assessed were significantly high, and the plaque index measured using QLF-D can be used as an assessment tool for not only evaluating gingival status objectively, but also for educating patients about plaque control and continued patient oral care. Therefore, this method can be an alternative for the conventional measurement of plaque depostion, moreover, it is considered that it can also be used for a large scale clinical examination to reduce the time and cost.

## Additional files

Additional files 1: Study description and consent form in Korean and English, respectively. Thirty-three adult patients aged over 20 years participated in the study and recruited from among the outpatients who visited the Ewha Womans University Mokdong Hospital, agreed to the terms of the research. (ZIP 49 kb)

Additional files 2: Study questionnaire in Korean and English, respectively. Self-reporting questionnaire was written by the participants, and included the following contents: sex, age, occupation, existence of systemic disease, use of toothbrush and other oral hygiene products, periodic scaling, and smoking status. (ZIP 26 kb)

Additional file 3: Results of measurement. All the datasets that were examined in this study including the QLF-D score, QLF-D ΔR 30, 70, 120 scores, and clinical indices such as GI, BOP, PPD, and PHP index are presented. All the measures was performed comparing the indices of maxillary and mandibular teeth, the teeth on right and left sides of the mouth, anterior and posterior teeth, and buccal and lingual surfaces of each tooth. (XLSX 289 kb)

## Abbreviations

BOP: Bleeding on probing
GI: Gingival index
PHP: Patient Hygiene Performance
PPD: Probing pocket depth
QLF-D: quantitative light-induced fluorescence-digital
SPS: Simple Plaque Score
