# Supplementary material for: Validity assessment of quantitative light-induced fluorescence-digital (QLF-D) for the dental plaque scoring system: a cross-sectional study
Source: BMC Oral Health. 2018 Nov 20;18:187. doi: 10.1186/s12903-018-0654-8 (PMC6247760; doi:10.1186/s12903-018-0654-8)
Supplement: Supplementary file 1 — Study description and consent form in Korean and English, respectively. Thirty-three adult patients aged over 20 years participated in the study and recruited from among the outpatients who visited the Ewha Womans University Mokdong Hospital, agreed to the terms of the research. (ZIP 49 kb) [file 12903_2018_654_MOESM1_ESM.zip › JongBinLee_Additional-File1-1_[Consent-Form]_KORR3.doc]

¿¬±¸´ë»óÀÚ ¼³¸í¹® ¹× µ¿ÀÇ¼­
(ÀÌ´ë¸ñµ¿º´¿ø)
¿¬±¸°úÁ¦¸í	Quantitative Light Induced Fluorescence Digital (QLF-D)À» ÀÌ¿ëÇÑ Ä¡¸é¼¼±Õ¸· °Ë»ç¹ýÀÇ ÀÓ»óÀû È°¿ë	
¿¬±¸Ã¥ÀÓÀÚ	(¼º¸í)	¹æÀº°æ	(¼Ò¼Ó)	ÀÌÈ­¿©ÀÚ´ëÇÐ±³ ÀÓ»óÄ¡ÀÇÇÐ´ëÇÐ¿ø	(¿¬¶ôÃ³)	02-2650-2679/ 02-2650-2660	
¿¬±¸´ã´çÀÚ	(¼º¸í)	ÃÖ´ÙÇý	(¼Ò¼Ó)	ÀÌÈ­¿©ÀÚ´ëÇÐ±³ ÀÓ»óÄ¡ÀÇÇÐ´ëÇÐ¿ø	(¿¬¶ôÃ³)	010-3088-9822	

ÀÌ ¿¬±¸´Â Quantitative Light Induced Fluorescence Digital (QLF-D)À» ÀÌ¿ëÇÑ Ä¡¸é¼¼±Õ¸· °Ë»ç¹ýÀÇ ÀÓ»óÀû È°¿ë¿¡ ´ëÇÑ ¿¬±¸ÀÔ´Ï´Ù. ±ÍÇÏ´Â ¿¬±¸ ¼öÇà¿¡ ÀûÇÕÇÑ ÇÇÇèÀÚÀÌ±â ¶§¹®¿¡ ÀÌ ¿¬±¸¿¡ Âü¿©ÇÏµµ·Ï ±ÇÀ¯ ¹Þ¾Ò½À´Ï´Ù. ÀÌ ¿¬±¸¸¦ ¼öÇàÇÏ´Â ÀÌ´ë¸ñµ¿º´¿ø ¼Ò¼Ó ¹æÀº°æ(Ã¥ÀÓ¿¬±¸ÀÚ/02-2650-2679)ÀÌ ±ÍÇÏ¿¡°Ô ¿¬±¸ Âü¿© °úÁ¤¿¡ ´ëÇÏ¿© ¼³¸íÇØ ÁÙ °ÍÀÔ´Ï´Ù.
 ÀÌ ¿¬±¸´Â ÀÚ¹ßÀûÀ¸·Î Âü¿© ÀÇ»ç¸¦ ¹àÈ÷½Å ºÐ¿¡ ÇÑÇÏ¿© ¼öÇà µÉ °ÍÀÌ¸ç, ±ÍÇÏ²²¼­´Â º» ÀÓ»ó ¿¬±¸¿¡ Âü¿© ÀÇ»ç¸¦ °áÁ¤ÇÏ±â¿¡ ¾Õ¼­, º» ÀÓ»ó¿¬±¸°¡ ¿Ö ¼öÇàµÇ°í, ±ÍÇÏÀÇ Á¤º¸°¡ ¾î¶»°Ô »ç¿ëµÉÁö, º» ÀÓ»ó¿¬±¸°¡ ¾î¶² °ÍÀ» Æ÷ÇÔÇÏ°í ÀÖ´Â Áö¿Í °¡´ÉÇÑ ÀÌÁ¡, À§Çè, ºÒÆíÇÔÀº ¹«¾ùÀÎÁö¿¡ ´ëÇÏ¿© ÀÌÇØÇÏ´Â °ÍÀÌ Áß¿äÇÕ´Ï´Ù.
´ÙÀ½ÀÇ ¼³¸íÀ» ½ÅÁßÇÏ°Ô ½Ã°£À» °¡Áö°í ÁÖÀÇ ±í°Ô ÀÐÀ¸½Ã±â ¹Ù¶ó¸ç, ÇÊ¿äÇÏ½Ã¸é ±ÍÇÏÀÇ ÁÖÄ¡ÀÇ ¶Ç´Â °¡Á·ÀÌ³ª Ä£±¸µé°ú »óÀÇÇÏ½Ã±â ¹Ù¶ø´Ï´Ù. ¸¸ÀÏ ¾î¶°ÇÑ Áú¹® »çÇ×ÀÌ ÀÖÀ¸½Ã¸é ´ã´ç ¿¬±¸ÀÚ°¡ ÀÚ¼¼ÇÏ°Ô ¼³¸íÇØ ÁÙ °ÍÀÔ´Ï´Ù.
±ÍÇÏÀÇ ¼­¸íÀº ±ÍÇÏ°¡ º» ¿¬±¸¿¡ ´ëÇØ ±×¸®°í ¿¬±¸Âü¿©¿¡ µû¸¥ À§Çè¼º¿¡ ´ëÇØ ¼³¸íÀ» µè°í ÀÌÇØÇßÀ½À» ÀÇ¹ÌÇÏ¿©, ±ÍÇÏ²²¼­(¶Ç´Â ¹ýÁ¤´ë¸®ÀÎ) º» ¿¬±¸¿¡ Âü¿©¸¦ ¿øÇÑ´Ù´Â °ÍÀ» ÀÇ¹ÌÇÕ´Ï´Ù.


1. ¿¬±¸ÀÇ ¹è°æ°ú ¸ñÀû
 º» ¿¬±¸¿¡ È°¿ëµÇ´Â ÀÇ·á±â±â Quantitative Light induced Fluorescence-Digital(QLF-D) device´Â ÄÄÇ»ÅÍ ±â¹Ý Ä¡ÅÂ¸éÀûÃøÁ¤¹ýÀÇ ÇÏ³ª·Î¼­, ±¸°­ ³» ¼¼±ÕÀÌ »ý»êÇÏ´Â Æ÷ÇÇ¸°¿¡ ÀÇÇØ ³ªÅ¸³ª´Â ºÓÀº Çü±¤ÀÇ Ä¡ÅÂ¸¦ Å½ÁöÇÏ´Â ÀåºñÀÌ¸ç ÀÛÀº ¾çÀÇ Ä¡ÅÂ º¯È­¸¦ °´°üÀûÀ¸·Î Å½ÁöÇÒ ¼ö ÀÖ´Â ÀÇ·á±â±âÀÔ´Ï´Ù. ´Ù¸¥ ÄÄÇ»ÅÍ ±â¹Ý Ä¡ÅÂÃøÁ¤¹ýÀº Ä¡¸éÂø»öÁ¦¸¦ ÀÌ¿ëÇØ Ä¡ÅÂ¸¦ ½Äº°ÇÏ´Â ¹Ý¸é, QLF´Â º°µµÀÇ Ä¡¸é Âø»öÀÌ ÇÊ¿ä ¾ø´Â ÀåÁ¡ÀÌ ÀÖ½À´Ï´Ù. ±×·¯³ª ¾ÆÁ÷±îÁö »õ·Î¿î Ä¡ÅÂÃøÁ¤¹ýÀ¸·Î¼­ QLF-D¸¦ ÀÌ¿ëÇÑ Ä¡ÅÂ¸éÀûÃøÁ¤¹ý¿¡ ´ëÇÑ ¿¬±¸°¡ È°¹ßÈ÷ ÀÌ·ç¾îÁö°í ÀÖÁö ¾ÊÀº ½ÇÁ¤ÀÔ´Ï´Ù.
 ±×·¯¹Ç·Î º» ¿¬±¸¿¡¼­´Â »õ·Ó°Ô °³¹ßµÈ ±¤ÇÐ ÀåºñÀÎ QLF-D ÀÇ·á±â±â¸¦ ÀÌ¿ëÇÑ Ä¡¸é¼¼±Õ¸· °Ë»ç¹ýÀ» È°¿ëÇÏ¿© Ä¡ÅÂ¸éÀûÃøÁ¤¹ý°ú ÀÕ¸ö°Ç°­ »óÅÂ¿¡ ´ëÇÑ ¿¬°ü¼ºÀ» Æò°¡ÇÏ´Â µ¥¿¡ ÀÖ½À´Ï´Ù. 	

2. ¿¬±¸ Âü¿© ´ë»ó
º» ¿¬±¸¿¡´Â ¼ºÀåÀÌ ¿Ï·á µÇ°í »ó,ÇÏ¾Ç ÀüÄ¡ºÎ ,±¸Ä¡ºÎ Ä¡¿­ÀÌ ºñ±³Àû °í¸¥ 20¼¼ ÀÌ»óÀÇ ¼ºÀÎÀ» ´ë»óÀ¸·Î ¿¬±¸°¡ ÁøÇàµË´Ï´Ù.

º» ¿¬±¸ÀÇ ¸ñÇ¥ ÇÇÇèÀÚ ¼ö´Â 40¸íÀÔ´Ï´Ù.	

3. ÀÇ·á±â±â¿¡ ´ëÇÑ Á¤º¸ 
QLF-D Biluminator2´Â Canon 450D ÀÌ»óÀÇ DSLR Ä«¸Þ¶ó¸¦ ±âº»À¸·Î ÇÏ´Â ÀåºñÀÔ´Ï´Ù.
À°¾ÈÀ¸·Î È®ÀÎÇÏ±â ¾î·Á¿î ÃÊ±â ÃæÄ¡(incipient caries), Ä¡ÅÂ(plaque), Ä¡¼®(calculus), Ä¡¾Æ ÆÄÀý(fracture), Ä¡¾Æ ±Õ¿­(crack) µîÀ» ºÓÀº »öÀÇ Çü±¤À¸·Î ½±°Ô È®ÀÎÇÒ ¼ö ÀÖµµ·Ï ÇØÁÖ¸ç, ÃÔ¿µµÈ Å¥·¹ÀÌ ¿µ»ó°ú ÀÏ¹Ý ¿µ»óÀ» Á¡¼ö¿Í ±×·¡ÇÁ·Î ºÐ¼®ÇØÁÖ´Â ¼¼°èÀûÀÎ ¿¬±¸¿ë ÀåºñÀÔ´Ï´Ù.	

4. ¿¬±¸¹æ¹ý ¹× ÀýÂ÷
±ÍÇÏ²²¼­ º» ¿¬±¸¿¡ Âü¿©ÇÏ½Ã±â·Î °áÁ¤ÇÏ¼Ì´Ù¸é, µ¿ÀÇ¼­ ¾ç½Ä¿¡ ¼­¸íÇÏ½Ã°Ô µË´Ï´Ù. ±ÍÇÏÀÇ ÀÚ¹ßÀûÀÎ Âü¿©ÀÇ»ç·Î ¿¬±¸°¡ ÁøÇàµÇ°Ô µË´Ï´Ù.
º» ÀÓ»ó½ÃÇè¿¡¼­´Â ÇÇÇèÀÚÀÇ ÀÏ¹ÝÀûÀÎ Æ¯¼ºÀ» Á¶»çÇÏ±â À§ÇÏ¿© ¼³¹®Á¶»ç°¡ ½ÃÇàµË´Ï´Ù. ±× ÈÄ ±¸³» ºÎÀ§º° Ä¡¾ÆÀÇ »çÁøÀ» QLF-D ÀÇ·á±â±â¸¦ ÀÌ¿ëÇÏ¿© ÃÑ 12ºÎÀ§ ÃÔ¿µÇÏ°Ô µË´Ï´Ù. ÃÔ¿µ µÈ »çÁøÀ¸·Î Ä¡¾Æ¿¡ ºÎÂøµÇ¾îÀÖ´Â Ä¡¸é¼¼±Õ¸· Áö¼ö¸¦ ºÐ¼®ÇÁ·Î±×·¥À» ÀÌ¿ëÇÏ¿© »êÃâÇÕ´Ï´Ù.
»çÁø ÃÔ¿µÀÌ ¿Ï·á µÈ ÈÄ ¼÷·ÃµÈ °Ë»çÀÚ°¡ ÇÇÇèÀÚÀÇ ±¸°­»óÅÂ¿¡ ´ëÇÑ °Ë»ç¸¦ ½Ç½ÃÇÕ´Ï´Ù. ±¸°­»óÅÂ¿¡ ´ëÇÑ °Ë»ç¿¡´Â ÀÕ¸ö °Ç°­ Æò°¡¿Í ÀÕ¸ö ±íÀÌ ÃøÁ¤, Å½Ä§ ÈÄ ÀÕ¸ö ÃâÇ÷¿©ºÎ¿¡ ´ëÇÑ Æò°¡°¡ ½Ç½ÃµË´Ï´Ù.
±¸°­ »óÅÂ¿¡ ´ëÇÑ °Ë»ç ÈÄ Ä¡¾Æ¿¡ ºÙ¾îÀÖ´Â Ä¡¸é¼¼±Õ¸·ÀÇ ¾çÀ» Æò°¡ÇÏ±â À§ÇÏ¿© ¿°»öÁ¦¸¦ ÀÌ¿ëÇÏ¿© Ä¡¸é¼¼±Õ¸· ¿°»ö ÈÄ ±¸°­ ³» Ä¡¾Æ 12ºÎÀ§ ÃÔ¿µÇÏ°Ô µË´Ï´Ù.
 º» ¿¬±¸·Î ¾ò¾îÁø µ¥ÀÌÅÍ´Â QLF-D ÀÇ·á±â±â¸¦ ÀÌ¿ëÇÑ Ä¡¸é¼¼±Õ¸· ºÐ¼®°ú °Ë»çÀÚ°¡ À°¾ÈÀ¸·Î È®ÀÎÇÏ´Â Ä¡¸é¼¼±Õ¸· Áö¼öÀÇ ¿¬°ü¼º¿¡ ´ëÇÑ Æò°¡¿Í QLF-D Ä¡¸é¼¼±Õ¸· Áö¼ö¿Í ÀÕ¸ö°Ç°­°úÀÇ ¿¬°ü¼ºÀÌ ÀÖ´ÂÁö¸¦ Æò°¡ÇÏ±â À§ÇÏ¿© ºÐ¼®¿¡ È°¿ëµË´Ï´Ù.	

5. ¿¬±¸ ¼Ò¿ä ½Ã°£
1) ÇÇÇèÀÚÀÇ ÀÏ¹ÝÀûÀÎ Æ¯¼ºÀ» Á¶»çÇÏ±â À§ÇÑ ¼³¹®Á¶»ç ½ÃÇà (10ºÐ ¼Ò¿ä)
2) ±¸³» ºÎÀ§º° »çÁø ÃÔ¿µ(15ºÐ ¼Ò¿ä)
-»ó.ÇÏ¾Ç ÁÂ.¿ì ±¸Ä¡ºÎ Çù¸é, »ó.ÇÏ¾Ç ÁÂ.¿ì ±¸Ä¡ºÎ ¼³¸é, »ó.ÇÏ¾Ç ÀüÄ¡ºÎ Çù.¼³¸é »çÁø ÃÔ¿µ.
-ÃÑ 12ºÎÀ§.
3) ±¸°­ »óÅÂ °Ë»ç (20ºÐ ¼Ò¿ä)
¡¤ÀÕ¸ö ±íÀÌ ÃøÁ¤
¡¤ÀÕ¸ö °Ç°­ »óÅÂ Æò°¡
¡¤Ä¡¾Æ Ä¡¸é¼¼±Õ¸· Áö¼ö Æò°¡
4) Ä¡¾Æ Ä¡¸é Âø»ö ÈÄ ºÎÀ§º° ±¸°­ »çÁø ÃÔ¿µ (15ºÐ ¼Ò¿ä)

º» ¿¬±¸¸¦ ¼öÇàÇÏ´Âµ¥ ¼Ò¿äµÇ´Â ½Ã°£Àº ¾à 1½Ã°£ ³»¿ÜÀÔ´Ï´Ù.	

6. ¿¬±¸ Âü¿© µµÁß ÁßµµÅ»¶ô
 ¿¬±¸¿¡ Âü°¡ÇÏ´Â °ÍÀº ÀÚÀÇÀÌ¸ç ÇÇÇèÀÚ ÀÚ½ÅÀÌ ÀÚ¹ßÀûÀ¸·Î Âü¿©¸¦ °áÁ¤ÇÒ ¼ö ÀÖ½À´Ï´Ù. ¸¸ÀÏ Âü¿©¿¡ µ¿ÀÇÇÏÁö ¾Ê´õ¶óµµ ÀüÇô ºÒÀÌÀÍÀ» ¹ÞÁö ¾Ê½À´Ï´Ù.
¶ÇÇÑ, ÀÓ»ó½ÃÇè Âü¿©¸¦ Áß´ÜÇÏ½Ã´õ¶óµµ °è¼ÓÇØ¼­ º´¿ø¿¡¼­ Ä¡·á¸¦ ¹ÞÀ¸½Ç ¼ö ÀÖÀ¸¸ç, ±ÍÇÏÀÇ ÀÇÇÐÀû Ä¡·á¿¡´Â ¿µÇâÀÌ ¾øÀ» °ÍÀÔ´Ï´Ù. ÀÓ»ó½ÃÇè Âü¿©·Î ÀÎÇÑ ¼Õ»ó ¹ß»ý½Ã, ±ÍÇÏ²²´Â ¼Õ»ó¿¡ ´ëÇÑ º¸»óÀ» ¹ÞÀ» ±Ç¸®°¡ ÀÖ½À´Ï´Ù.
±ÍÇÏ´Â º» ÇÇÇèÀÚ µ¿ÀÇ ¼³¸í¼­¸¦ ¸ðµÎ ÀÐ°í ÀÌÇØÇÑ ÈÄ, ±ÍÇÏÀÇ ¸ðµç ÀÇ¹®Á¡¿¡ ´ëÇØ ÀÓ»ó¿¬±¸ÀÚÀÇ ¸¸Á·½º·¯¿î ´äº¯À» µéÀº ÈÄ, º°µµ·Î ¸¶·ÃµÈ µ¿ÀÇ¼­¿¡ ¼­¸íÇÏ½Ã¸é µË´Ï´Ù. ÇÇÇèÀÚÀÇ Áú¹®¿¡ ´ëÇÑ ´ë´äÀÌ ¿Ïº®ÇÏ°Ô ÀÌ·ç¾îÁöÁö ¾Ê´Â ÇÑ, ¼­¸íÇÏÁö ¾ÊÀ¸¼Åµµ µË´Ï´Ù.	

7. ¿¬±¸Âü¿©·Î ÀÎÇØ ¿¹»óµÇ´Â À§Çè(ºÎÀÛ¿ë) ¹× ºÒÆí»çÇ×
±¸³» »çÁø ÃÔ¿µ ½Ã ÀÔ¼ú °ßÀÎÀ» À§ÇÑ °ßÀÎ±â Á¶ÀÛÀ¸·Î ÀÎÇÏ¿© ¼ø°£ÀûÀÎ ºÒÆíÇÔÀ» °æÇèÇÒ ¼ö ÀÖÀ¸³ª ÀÌ´Â °Ë»ç ÈÄ Áï½Ã »ç¶óÁý´Ï´Ù.
Ä¡ÁÖ³¶ ±íÀÌ ÃøÁ¤ ½Ã ÃøÁ¤ ±â±¸ Á¶ÀÛÀ¸·Î ÀÎÇÏ¿© ¹Ì¾àÇÑ ÅëÁõÀÌ ¹ß»ýÇÒ ¼ö ÀÖ½À´Ï´Ù.
¸¸ÀÏ ¿¬±¸Âü¿© µµÁß ¹ß»ý ÇÒ ¼ö ÀÖ´Â ºÎÀÛ¿ëÀÌ³ª ºÒÆí°¨¿¡ ´ëÇØ ±Ã±ÝÇÑ »çÇ×ÀÌ ÀÖÀ¸½Ã¸é ¾ðÁ¦µçÁö ¿¬±¸´ã´çÀÚ¿¡°Ô Áú¹®ÇÏ½Ã±â ¹Ù¶ø´Ï´Ù.	

8. ¿¬±¸ Âü¿©¿¡ µû¸¥ ÀÌÀÍ
º» ¿¬±¸¿¡ Âü¿©ÇÔÀ¸·Î¼­ ±ÍÇÏ¿¡°Ô ±â´ëµÇ´Â Á÷Á¢ÀûÀÎ ÇýÅÃÀº ¾ø½À´Ï´Ù.
±×·¯³ª º» ¿¬±¸¸¦ ÅëÇØ »õ·Î¿î Ä¡¸é¼¼±Õ¸· °Ë»çÀåÄ¡ÀÇ »ç¿ë¼ºÀÌ ÀÔÁõµÇ¸é ÇâÈÄ È¿À²ÀûÀÎ ÀÓ»ó±â±â ¹ßÀüÀ» ±â´ëÇÒ ¼ö ÀÖ½À´Ï´Ù.	

9. ±ÝÀüÀû Áö±Þ ¶Ç´Â ¿¬±¸¿¡ Âü¿©ÇÔÀ¸·Î½á Ãß°¡ÀûÀ¸·Î ¹ß»ýµÇ´Â ºñ¿ë
º» ¿¬±¸ Âü¿©·Î ÀÎÇØ ±ÍÇÏ¿¡°Ô Áö±Þ µÇ´Â ±ÝÀüÀû ÇýÅÃÀº ¾øÀ¸³ª ¼ÒÁ¤ÀÇ ±â³äÇ°À» µå¸³´Ï´Ù. ¶ÇÇÑ ±ÍÇÏ¿¡°Ô Ãß°¡ÀûÀ¸·Î ¹ß»ýÇÏ´Â º°µµÀÇ ºñ¿ëÀº ¾ø½À´Ï´Ù.	

10. ÀÚ¹ßÀû Âü¿©
º» ÀÓ»ó½ÃÇè¿¡ Âü¿©ÇÏ½Ã´Â °ÍÀº ±ÍÇÏ¿¡°Ô ´Þ·Á ÀÖ½À´Ï´Ù. ±ÍÇÏ´Â ¾ðÁ¦µçÁö ½ÃÇè¿¡ Âü¿©ÇÏÁö ¾Ê±â·Î °áÁ¤ÇÒ ¼ö ÀÖ°í ¶ÇÇÑ ½ÃÇèÀ» ±×¸¸ µÑ ¼ö ÀÖ½À´Ï´Ù.
º» ¿¬±¸¿¡ Âü¿©ÇÏÁö ¾Ê¾Æµµ ¾Æ¹«·± ºÒÀÌÀÍÀ» ¹ÞÁö ¾ÊÀ¸¸ç ±ÍÇÏÀÇ °áÁ¤Àº Çâ ÈÄ ±ÍÇÏ°¡ Áø·á¸¦ ¹Þ´Â °Í¿¡ ¿µÇâÀ» ¹ÌÄ¡Áö ¾Ê½À´Ï´Ù.	

11. °³ÀÎÁ¤º¸¿Í ºñ¹Ð º¸Àå
 º» ¿¬±¸ÀÇ Âü¿©·Î ÀÎÇØ ±ÍÇÏ¿¡°Ô¼­ ¼öÁýµÇ´Â °³ÀÎÁ¤º¸´Â ´ÙÀ½°ú °°½À´Ï´Ù. (¼ºº°, Á÷¾÷, ³ªÀÌ, ±¸°­ »çÁø) ÀÌ Á¤º¸´Â ¿¬±¸¸¦ À§ÇØ 5³â°£ »ç¿ëµÇ¸ç ¼öÁýµÈ Á¤º¸´Â °³ÀÎÁ¤º¸º¸È£¹ý¿¡ µû¶ó ÀûÀýÈ÷ °ü¸®µË´Ï´Ù. °ü·Ã Á¤º¸´Â ¿¬±¸Ã¥ÀÓÀÚ ¹× °øµ¿¿¬±¸ÀÚ ¸¸ÀÌ Á¢±Ù °¡´ÉÇÕ´Ï´Ù.
ÀÌ ¿¬±¸¿¡¼­ ¾ò¾îÁø °³ÀÎÁ¤º¸°¡ ÃâÆÇµÇ°Å³ª ÇÐÈ¸Áö¿¡ °ø°³µÉ °æ¿ì ±ÍÇÏ¸¦ ½Äº°ÇÒ ¼ö ÀÖ´Â °³ÀÎÁ¤º¸´Â Áö¿öÁø Ã¤ Á¦°øµÉ °ÍÀÔ´Ï´Ù. ±×·¯³ª ¸¸ÀÏ ¹ýÀÌ ¿ä±¸ÇÏ¸é ±ÍÇÏÀÇ °³ÀÎÁ¤º¸´Â Á¦°øµÉ ¼öµµ ÀÖÀ¸¸ç ¸ð´ÏÅÍ ¿ä¿ø, Á¡°Ë¿ä¿ø, ±â°ü»ý¸íÀ±¸®½ÉÀÇÀ§¿øÈ¸´Â ¿¬±¸´ë»óÀÚÀÇ ºñ¹Ðº¸ÀåÀ» Ä§ÇØÇÏÁö ¾Ê°í °ü·Ã±ÔÁ¤ÀÌ Á¤ÇÏ´Â ¹üÀ§ ³»¿¡¼­ ±ÍÇÏ¸¦ ½Äº°ÇÒ ¼ö ÀÖ´Â µ¥ÀÌÅÍ¸¦ ¿­¶÷ÇÒ ¼ö ÀÖ½À´Ï´Ù. ±ÍÇÏ°¡ º» µ¿ÀÇ¼­¿¡ ¼­¸íÇÏ´Â °ÍÀº ÀÌ·¯ÇÑ »çÇ×¿¡ ´ëÇØ »çÀü¿¡ ¾Ë°í ÀÖ¾úÀ¸¸ç ÀÌ¸¦ Çã¿ëÇÑ´Ù´Â ÀÇ»ç·Î °£ÁÖµÉ °ÍÀÔ´Ï´Ù. ¿¬±¸°ü·Ã ÀÚ·á´Â ¿¬±¸ Á¾·á ÈÄ 5³â°£ º¸°üµÇ¸ç ÀÌÈÄ Æó±âµÉ °ÍÀÔ´Ï´Ù.	

12. ¿¬±¸°ü·Ã ¹®ÀÇ ¹× ¿¬±¸´ë»óÀÚ ±ÇÀÍ¿¡ °üÇÑ Á¤º¸ Á¦°ø
¿¬±¸¿Í °ü·ÃµÇ¾î Ãß°¡ÀûÀÎ Á¤º¸¸¦ ¾ò°íÀÚ ÇÏ°Å³ª ¿¬±¸ ¹× ¿¬±¸¿Í °ü·ÃÀÌ ÀÖ´Â ¼Õ»óÀÌ ¹ß»ýÇÑ °æ¿ì ´ÙÀ½ ¿¬±¸´ã´çÀÚ¿¡°Ô ¾ðÁ¦µçÁö ¿¬¶ôÇÏ½Ê½Ã¿À

-ÀÓ»ó½ÃÇè Ã¥ÀÓÀÚ: ÀÌÈ­¿©ÀÚ´ëÇÐ±³ ÀÇÇÐÀü¹®´ëÇÐ¿ø Ä¡°úÇÐ±³½Ç ±³¼ö ¹æÀº°æ (¿¬¶ôÃ³: 02-2650-2679 / 02-2650-2660)
-ÀÓ»ó½ÃÇè ´ã´çÀÚ: ÀÌÈ­¿©ÀÚ´ëÇÐ±³ ÀÓ»óÄ¡ÀÇÇÐ´ëÇÐ¿ø ´ëÇÐ¿ø»ý ÃÖ´ÙÇý (¿¬¶ôÃ³: 010-3088-9822)

¶ÇÇÑ ±ÍÇÏ´Â ¿¬±¸ ´ë»óÀÚ·ÎÀÇ ±ÍÇÏÀÇ ±Ç¸®¿¡ ´ëÇØ ÀÇ¹®ÀÌ ÀÖÀ» °æ¿ì ±â°ü»ý¸íÀ±¸®½ÉÀÇÀ§¿øÈ¸ (02-2650-5872)·Î ¹®ÀÇÇÏ½Ç ¼ö ÀÖ½À´Ï´Ù.	
 
13. ÀÀ±Þ»çÇ× ¹ß»ý ½Ã 24½Ã°£ ¿¬¶ô °¡´ÉÇÑ ¿¬±¸ÀÚ ¿¬¶ôÃ³ 
º» ¿¬±¸ ÁøÇà ÈÄ ±ä±ÞÀ¸·Î ¹ß»ýÇÏ´Â ¹®Á¦¿¡ ´ëÇÑ ¹®ÀÇ´Â ¾Æ·¡ ¿¬¶ôÃ³·Î ¾ðÁ¦µçÁö ¿¬¶ôÇÏ½Ã±â ¹Ù¶ø´Ï´Ù.
-ÀÓ»ó½ÃÇè ´ã´çÀÚ: ÃÖ´ÙÇý (¿¬¶ôÃ³: 010-3088-9822)	


¿¬±¸´ë»óÀÚ µ¿ÀÇ¼­ (Version No.:       )
¿¬±¸°úÁ¦¸í	Quantitative Light Induced Fluorescence Digital (Q-RAY)À» ÀÌ¿ëÇÑ Ä¡¸é¼¼±Õ¸· °Ë»ç¹ýÀÇ ÀÓ»óÀû È°¿ë	
¿¬±¸Ã¥ÀÓÀÚ	(¼º¸í)	¹æÀº°æ	(¼Ò¼Ó)	ÀÌÈ­¿©ÀÚ´ëÇÐ±³
ÀÓ»óÄ¡ÀÇÇÐ´ëÇÐ¿ø	(¿¬¶ôÃ³)	010-6236-8478	

¡Ø ☑ Ç¥½Ã ¿äÇÔ.
1. º»ÀÎÀº ÀÓ»ó¿¬±¸¿¡ ´ëÇØ ±¸µÎ·Î ¼³¸íÀ» ¹Þ°í »ó±â ÇÇÇèÀÚ ¼³¸í¹®À» ÀÐ¾úÀ¸¸ç ´ã´ç ¿¬±¸ÀÚ¿Í ÀÌ¿¡ ´ëÇÏ¿© ÀÇ³íÇÏ¿´½À´Ï´Ù	☐	
2. º»ÀÎÀº À§Çè°ú ÀÌµæ¿¡ °üÇÏ¿© µé¾úÀ¸¸ç ³ªÀÇ Áú¹®¿¡ ¸¸Á·ÇÒ ¸¸ÇÑ ´äº¯À» ¾ò¾ú½À´Ï´Ù.	☐	
3. º»ÀÎÀº ÀÌ ¿¬±¸¿¡ Âü¿©ÇÏ´Â °Í¿¡ ´ëÇÏ¿© ÀÚ¹ßÀûÀ¸·Î µ¿ÀÇÇÕ´Ï´Ù.	☐	
4. º»ÀÎÀº ÀÌÈÄÀÇ Ä¡·á¿¡ ¿µÇâÀ» ¹ÞÁö ¾Ê°í ¾ðÁ¦µçÁö ¿¬±¸ÀÇ Âü¿©¸¦ °ÅºÎÇÏ°Å³ª ¿¬±¸ÀÇ Âü¿©¸¦ Áßµµ¿¡ Ã¶È¸ÇÒ ¼ö ÀÖ°í ÀÌ·¯ÇÑ °áÁ¤ÀÌ ³ª¿¡°Ô ¾î¶°ÇÑ ÇØ°¡ µÇÁö ¾ÊÀ» °ÍÀÌ¶ó´Â °ÍÀ» ¾Ë°í ÀÖ½À´Ï´Ù.	☐	
5. º»ÀÎÀº ÀÌ ¼³¸í¹® ¹× µ¿ÀÇ¼­¿¡ ¼­¸íÇÔÀ¸·Î½á ÀÇÇÐ ¿¬±¸ ¸ñÀûÀ¸·Î ³ªÀÇ °³ÀÎÁ¤º¸°¡ ÇöÇà ¹ý·ü°ú ±ÔÁ¤ÀÌ Çã¿ëÇÏ´Â ¹üÀ§ ³»¿¡¼­ ¿¬±¸ÀÚ°¡ ¼öÁýÇÏ°í Ã³¸®ÇÏ´Âµ¥ µ¿ÀÇÇÕ´Ï´Ù.	☐	
6. º»ÀÎÀº ÀÌ µ¿ÀÇ¼­ »çº»1ºÎ¸¦ ¹ÞÀ» °ÍÀ» ¾Ë°í ÀÖ½À´Ï´Ù.	☐	

ÇÇ Çè ÀÚ :	(¼º¸í)	(¼­¸í)	(¼­¸íÀÏ)	
µ¿ÀÇ¼­¸¦ ¼³¸íÇÑ »ç¶÷ :	(¼º¸í) 	(¼­¸í)	(¼­¸íÀÏ)	
¿¬±¸Ã¥ÀÓÀÚ	(¼º¸í) 	(¼­¸í)	(¼­¸íÀÏ)	
¿¬±¸´ã´çÀÚ	(¼º¸í) 	(¼­¸í)	(¼­¸íÀÏ)	
¹ýÀû ´ë¸®ÀÎ(ÇÊ¿ä ½Ã) :	(¼º¸í)	(¼­¸í)	(¼­¸íÀÏ)	
¹ýÀû ´ë¸®ÀÎ(ÇÊ¿ä ½Ã) :	(ÇÇÇèÀÚ¿ÍÀÇ °ü°è)			
ÀÔÈ¸ÀÎ(ÇÊ¿ä ½Ã) :	(¼º¸í) 	(¼­¸í)	(¼­¸íÀÏ)	
