# Supplementary material for: Validity assessment of quantitative light-induced fluorescence-digital (QLF-D) for the dental plaque scoring system: a cross-sectional study
Source: BMC Oral Health. 2018 Nov 20;18:187. doi: 10.1186/s12903-018-0654-8 (PMC6247760; doi:10.1186/s12903-018-0654-8)
Supplement: Supplementary file 1 — Study description and consent form in Korean and English, respectively. Thirty-three adult patients aged over 20 years participated in the study and recruited from among the outpatients who visited the Ewha Womans University Mokdong Hospital, agreed to the terms of the research. (ZIP 49 kb) [file 12903_2018_654_MOESM1_ESM.zip › JongBinLee_Additional-File1-2_[Consent-Form]_ENGR3.rtf]

Informed Consent From for Medical Research
(Ewha Womans University Mokdong Hospital)
Title of the Study	Validity assessment of quantitative light-induced fluorescence-digital (QLF-D) for the dental plaque scoring system	
Chief of the Study	(Name)	Eun-Kyoung Pang	(Affiliation)	Graduate School of Clinical Dentistry, Ewha Womans University	(Tel)	02-2650-2679 / 02-2650-2660	
Researcher of study	(Name)	Da-Hye Choi	(Affiliation)	Graduate School of Clinical Dentistry, Ewha Womans University	(Tel)	010-3088-9822	

This research is to investigate the clinical application of dental plaque scoring system using Quantitative Light Induced Fluorescence Digital (QLF-D). You are an eligible subject for this research; thus encouraged to participate in Eun-Kyoung Pang (the chief of the study/02-2650-2679) from Ewha Womans University Mokdong Hospital will explain the research participation process.
This study will be conducted only for those who have voluntarily participated. Prior to deciding whether or not to participate, it is important to understand the purpose of this research, how your information will be used, what this research includes, and possible benefits, risk, and discomfort.
Please carefully read the following instructions; and if necessary, discuss with your physician, family, or friends. If you have any questions, the researcher in charge will explain in detail. 
Your signature means that you have heard and understood the risks involved in participating in this research, and that you (or your legal representative) want to participate.


1. Background and Purpose of the Research
The medical device used in this study, Quantitative Light induced Fluorescence-Digital (QLF-D) device, is one of computer-based plaque area measuring methods. It detects the fluorescent red plaque appearing by the porphyrin produced by oral bacteria, and can also objectively detect a small amount of change in plaque. Other computer-based plaque measuring methods use teeth surfaces colourant to identify plaque, whereas QLF has an advantage of not requiring it. However, there has not yet been brisk research on plaque area measuring method using QLF-D.
Therefore, the purpose of this study is to evaluate the correlation between measuring method and gingival health and plaque area measuring method using the newly developed optical device, QLF-D.	

2. Participants Selection
This study is conducted targeting adults of 20 and over, who have completed growth, and have relatively even set of teeth in all dentition.

The target number of the study subjects is 40.	

3. Information on the Medical Device 
QLF-D Biluminator2 is a device based on DSLR camera higher than Canon 450D.
This is a world-class medical research equipment that allows easy identification of incipient caries, plaque, calculus, fracture, and crack with red fluorescence, and analyzes the captured Q-ray images and general images with scores and graphs.	

4. Research Methods and Procedures
If you choose to participate, you will sign on the consent form. Your participation in this research is entirely voluntary.
There will be a questionnaire to investigate the study subject's general characteristics. After that, a total of 12 sections' pictures of the teeth will be taken using the medical device, QLF-D. The index of dental plaque attached on tooth will be computed using an analysis program and the captured images.
After the photo shoot is completed, a skilled examiner will inspect the oral condition of the subject. Examination of the oral condition includes gum health assessment, gum depth measurement, and whether gum bleeds after dental probing.
After the oral examination, dental plaque will be coloured by using colourant to evaluate the amount of dental plaque attached on the tooth. Then 12 areas will be photographed.
The data obtained in this study will be used to analyze 1) the correlation between the analysis of dental plaque using QLF-D and the dental plaque index that an examiner checks with the naked eye, and 2) the relationship between QLF-D dental plaque index and gum health.	

5. Duration
1) Questionnaire to obtain the subject's general characteristic (10 minutes)
2) oral photo shooting by part (15 minutes)
- Photo shooting of all posterior teeth buccal and lingual/palatal surfaces, and all anterior teeth labial and lingual/palatal surfaces.
-Total of 12. 
3) Oral condition examination (20 minutes)
¡¤ gum depth measurement
¡¤ gum health status assessment
¡¤ The index of dental plaque assessment
4) tooth surface coloring and photo shooing by parts (15 minutes)
The time required for the above process is approximately 1 hour.	

6. Withdrawal in the Middle of Participation
Your participation in this research is entirely voluntary, and it is your choice whether to participate or not.
You may change your mind later and stop participating even if you agreed earlier. Although you change your mind, all the services you receive at this clinic will continue and nothing will change. You have right to be reimbursed if any damage or loss occurs during the research participation.
You may sign on the separate consent form after reading and understanding all the information provided. All of your questions can be answered by clinical researchers. You do not have to sign unless the answers to your questions are not sufficient.	

7. Expected Risks (Side Effects) and Discomfort
When taking photos, you could experience discomfort on lips momentarily due to manipulation of a retractor, but it disappears right after the examination.
Measuring the depth of periodontal pocket can result minor pain due to manipulation of the measuring device.
If you have any questions about possible side effects or discomfort during participation, please feel free to contact the researcher.	

8. Benefits
There will be no direct/financial benefit to you even if participating in this research.
However, if the usability of the new dental plaque scoring system device is verified through this research, the development of effective clinical devices can be expected in the future.	

9. Reimbursements or Additional Cost occur due to the participation
You will not be given any financial gain, but will receive a small gift. In addition, there will be no additional cost to you.	

10. Right to Refuse or Withdraw
You do not have to take part in this research if you do not wish to do so. You may also stop participating in the research at any time you choose.
You may stop participating in the research at any time you wish without losing any of your rights as a patient here.	

11. Personal Information and Confidentiality
The information collected from you will be as follows: gender, occupation, age, and oral imaging. The information will be used for 5 years for the research and the collected information will be properly managed in accordance with the Personal Information Protection Act. The collected information will be put away and no one but the researchers will be able to see it.
If the personal information obtained from this study is published in journal, the information that is able to identify you will be wiped out and provided. However, if requested by law, your personal information may be provided. Monitor personnel, inspectors, and Institutional Bioethics Review Board can access information that can identify you, within the scope of the applicable regulation. You are deemed to be aware of this and to have accepted it by signing this consent form. Research data will be kept for 5 years after the end of the research, and will be discarded.	

12. Provision of Information about Research Inquiries and Rights and Interest of Research Subjects
If you are interested in obtaining additional information related to the research, or if any damage related to the research occurs, please contact the researchers down below at any time.

-Chief of study : Department of Dentistry, School of Medicine, Ewha Womans University, Associate Professor, Eun-Kyoung Pang (Tel: 02-2650-2679/ 02-2650-2660)
-Researcher of study : Graduate School of Clinical Dentistry, Ewha Womans University, Graduate Student, Da-Hye Choi (Tel: 010-3088-9822)

You may also contact the Institutional Review Board (02-2650-5872), if you have any questions about your rights as a study subject.	
 
13. Who to Contact in Case of Emergency 
If you have any questions about emergency after the research, do not hesitate to contact us at the number below.
-Researcher of study: Da-Hye Choi (Tel: 010-3088-9822)	


Consent Form (Version No. :       )
Title of the Study	Validity assessment of quantitative light-induced fluorescence-digital (QLF-D) for the dental plaque scoring system	
Chief of the Study	(Name)	Eun-Kyoung Pang	(Affiliation)	Graduate School of Clinical Dentistry, Ewha Womans University	(Tel)	010-6236-8478	

¡Ø ☑ Marking is required.
1. I have read the foregoing information and it has been explained to me, and discussed it with the researcher in charge. 	☐	
2. I have heard about the possible risks and benefits. I have also had the opportunity to ask questions about it and any questions that I have asked have been answered to my satisfaction.	☐	
3. I consent voluntarily to participate as a participant in this research.	☐	
4. I understand that I can refuse or withdraw the participation in this research at any time, and that this decision will not affect any harm to me.	☐	
5. I agree that researchers' collecting and processing of my personal information to the extent permitted by current laws and regulations for medical research purposes by signing this Informed Consent Form.	☐	
6. A copy of this Informed Consent Form has been provided to the participant.	☐	

Participant:	(Print Name)	(Signature)	(Date)	
Person who explained the consent:	(Print Name) 	(Signature)	(Date)	
Chief of the Research	(Print Name) 	(Signature)	(Date)	
Researcher in Charge	(Print Name) 	(Signature)	(Date)	
Legal representative (if necessary):	(Print Name)	(Signature)	(Date)	
¹ýÀû ´ë¸®ÀÎ(ÇÊ¿ä ½Ã) :	(Relation to the participant)			
Witness (if necessary):	(Print Name) 	(Signature)	(Date)	
