# Supplementary material for: Validity assessment of quantitative light-induced fluorescence-digital (QLF-D) for the dental plaque scoring system: a cross-sectional study
Source: BMC Oral Health. 2018 Nov 20;18:187. doi: 10.1186/s12903-018-0654-8 (PMC6247760; doi:10.1186/s12903-018-0654-8)
Supplement: Supplementary file 2 — Study questionnaire in Korean and English, respectively. Self-reporting questionnaire was written by the participants, and included the following contents: sex, age, occupation, existence of systemic disease, use of toothbrush and other oral hygiene products, periodic scaling, and smoking status. (ZIP 26 kb) [file 12903_2018_654_MOESM2_ESM.zip › JongBinLee_Additional-File2-1_[Questionnaire]_KORR3.doc]

¼³ ¹® Áö	

º» ¼³¹®Áö´Â Quantitative Light Induced Fluorescence Digital (Q-RAY)À» ÀÌ¿ëÇÑ Ä¡¸é¼¼±Õ¸· °Ë»ç¹ýÀÇ ÀÓ»óÀû È°¿ë ¿¬±¸¿¡ °üÇÑ ±âÃÊÀÚ·á¸¦ ¾ò°íÀÚ ÀÛ¼ºµÈ °ÍÀÔ´Ï´Ù.  º» ¼³¹®ÁöÀÇ ÀÀ´ä¿¡´Â Á¤․¿À´äÀÌ µû·Î ¾ø°í, Á¶»ç³»¿ëÀº ¿¬±¸¸ñÀû ÀÌ¿Ü¿¡´Â ¾î¶² ´Ù¸¥ ¸ñÀû¿¡µµ »ç¿ëµÇÁö ¾ÊÀ» °ÍÀÌ¸ç, ¸ðµç ÀÀ´ä³»¿ëÀº ÄÄÇ»ÅÍ·Î ±âÈ£È­ÇÏ¿© Ã³¸®µÇ¹Ç·Î Àý´ë ºñ¹ÐÀÌ º¸ÀåµË´Ï´Ù.  °¢ Áú¹®¿¡ ¼ÖÁ÷ÇÏ°Ô ÀÀ´äÇÏ¿© ÁÖ½Ã°í ÇÑ ¹®Ç×µµ ºüÁü¾øÀÌ ±âÀÔÇÏ¿© ÁÖ½Ã±â¸¦ ºÎÅ¹µå¸³´Ï´Ù.

ÀÌÈ­¿©ÀÚ´ëÇÐ±³ ÀÓ»óÄ¡ÀÇÇÐ´ëÇÐ¿ø ÃÖ´ÙÇý(010-3088-9822)	
¢À ´ÙÀ½ °¢ ¹°À½À» ÀÐÀ¸½Ã°í ÇØ´çÇÏ´Â °÷¿¡ ¡®¡î¡¯ ¶Ç´Â ¡®¡Û¡¯Ç¥¸¦ ÇØÁÖ½Ã±â ¹Ù¶ø´Ï´Ù.
1. ±ÍÇÏÀÇ ¼ºº°Àº?
(1) ³²ÀÚ  (2) ¿©ÀÚ
2. ±ÍÇÏÀÇ ¿¬·ÉÀº?
(1) 10´ë  (2) 20´ë  (3) 30´ë  (4) 40´ë (5) 50´ë (6) 60´ë ÀÌ»ó
3. ±ÍÇÏÀÇ Á÷¾÷Àº?
(1) ÇÐ»ý (2) »ç¹«Á÷ (3) »ý»êÁ÷ (4) ÀÇ·á°è Á¾»çÀÚ (5) ±âÅ¸(       )
4.´ÙÀ½°ú °°Àº ÁúÈ¯À» ¾Î°í ÀÖ°Å³ª ¾ÎÀº ÀûÀÌ ÀÖ½À´Ï±î?
                                       (ÇØ´ç ÁúÈ¯¿¡ Ã¼Å©ÇÏ¼¼¿ä.)
¡¤ ½ÉÀåº´                (           ) 
¡¤ ½ÉÀåº¸Á¶¹Úµ¿±â        (           ) 
¡¤ °íÇ÷¾Ð                (           ) 
¡¤ ³úÁ¹Áß                (           ) 
¡¤ ·ù¸¶Æ¼½º¿­            (           ) 
¡¤ ½ÅÀåÁúÈ¯              (           ) 
¡¤ °£¿°ÀÌ³ª °£ÁúÈ¯       (           ) 
¡¤ ´ç´¢                  (           ) 
¡¤ °áÇÙ                  (           ) 
¡¤ ¼ºº´                  (           ) 
¡¤ ºóÇ÷                  (           ) 
¡¤ Ç÷¿ìº´                (           ) 

5. ´ã¹è¸¦ ÇÇ¿ì½Ê´Ï±î? (¿¹, ¾Æ´Ï¿À)
ÇÏ·ç Áß ÇÇ´Â ¾ç:       °©,       °³ÇÇ

6. ÃÖ±Ù¿¡ Ä¡°úÁø·á¸¦ ¹ÞÀ¸½Å Àû ÀÖ½À´Ï±î?
(1) 1°³¿ù ÀÌ³» (2) 1~6°³¿ù ÀÌ³» (3) 6°³¿ù~1³â ÀÌ³»  (4) 1³â ÀÌ»ó 

7. Ä¡°ú¸¦ ¹æ¹®ÇÏ¿© Á¤±âÀûÀ¸·Î ½ºÄÉÀÏ¸µÀ» ¹Þ°í ÀÖ½À´Ï±î?
(1) Á¤±âÀûÀ¸·Î ¹Þ°í ÀÖ´Ù.(7-1¿¡ ÀÀ´äÇÏ¿©ÁÖ¼¼¿ä.)
(2) Á¤±âÀûÀ¸·Î ¹Þ°í ÀÖÁö ¾Ê´Ù.

7-1 Á¤±âÀûÀ¸·Î ½ºÄÉÀÏ¸µÀ» ¹Þ°í ÀÖ´Ù¸é ¾ó¸¶ ÁÖ±â·Î ¹Þ°í °è½Ê´Ï±î?
(1) 1°³¿ù~3°³¿ù (2) 4°³¿ù~6°³¿ù (3) 6°³¿ù~ 1³â (4) 1³â¿¡¼­ 2³â
(5) 2³â ÀÌ»ó

8. ÇÏ·ç Æò±Õ ÀÕ¼ÖÁú È½¼ö´Â ¸î ¹øÀÔ´Ï±î?
(1)ÇÏ·ç ÇÑ¹ø   (2)ÇÏ·ç µÎ ¹ø  (3) ÇÏ·ç ¼¼ ¹ø (4)ÇÏ·ç ³× ¹ø ÀÌ»ó

9. ÇÏ·ç Áß Ä©¼ÖÁú ½Ã±â¸¦ ¸ðµÎ ¡ýÇÏ¼¼¿ä.
(1)½ÄÈÄ¸¶´Ù (    )    (2)¾ÆÄ§½Ä»ç Àü (    )  (3)¾ÆÄ§½Ä»ç ÈÄ (    )
(4)Á¡½É½Ä»ç ÈÄ (    ) (5)Àú³á½Ä»ç ÈÄ (    ) 

10.Ä©¼ÖÀº ¸î °³¿ù¸¶´Ù ±³È¯ÇÕ´Ï±î? (      )°³¿ù

11.Ä©¼ÖÀ» Á¦¿ÜÇÑ ±× ¿Ü(Ä¡½Ç,Ä¡°£Ä©¼Ö,±¸°­¾çÄ¡¾×,ÇôÅ¬¸®³Ê µî)ÀÇ ±¸°­°ü¸®¿ëÇ°À» »ç¿ëÇÏ°í ÀÖ½À´Ï±î?
(1) »ç¿ëÇÏ°í ÀÖ´Ù.(11-1 ¼³¹®Áö¿¡ ÀÀ´äÇÏ¿©ÁÖ¼¼¿ä.)  (2) »ç¿ëÇÏÁö ¾Ê´Â´Ù.

11-1. ±¸°­À§»ý¿ëÇ° (ÀÌ¾¥½Ã°³ Á¦¿Ü)À» »ç¿ëÇÏ°í ÀÖÀ¸½Ã¸é ¾î¶² ±¸°­°ü¸®¿ëÇ°À» »ç¿ëÇÏ°íÀÖ½À´Ï±î?
(1) Ä¡½Ç    (2) Ä¡°£Ä©¼Ö   (3) ±¸°­¾çÄ¡¾×(°¡±×¸°,¸®½ºÅ×¸° µî)
(4) Çô Å¬¸®³Ê (5) ¿öÅÍ ÇÈ (6) ±âÅ¸ :(               ) 


 ¼³¹®¿¡ ÀÀÇØÁÖ¼Å¼­ °¨»çÇÕ´Ï´Ù.
