# Supplementary material for: Validity assessment of quantitative light-induced fluorescence-digital (QLF-D) for the dental plaque scoring system: a cross-sectional study
Source: BMC Oral Health. 2018 Nov 20;18:187. doi: 10.1186/s12903-018-0654-8 (PMC6247760; doi:10.1186/s12903-018-0654-8)
Supplement: Supplementary file 2 — Study questionnaire in Korean and English, respectively. Self-reporting questionnaire was written by the participants, and included the following contents: sex, age, occupation, existence of systemic disease, use of toothbrush and other oral hygiene products, periodic scaling, and smoking status. (ZIP 26 kb) [file 12903_2018_654_MOESM2_ESM.zip › JongBinLee_Additional-File2-2_[Questionnaire]_ENGR3.rtf]

Questionnaire	

 This questionnaire was prepared to obtain basic data on the clinical application of the dental plaque scoring system using Quantitative Light Induced Fluorescence Digital (Q-RAY). There is no correct answer in this questionnaire. The content will not be used for any purpose other than the purpose of research, and all responses will be encoded, thus ensured absolute confidentiality. Please answer frankly to each question and fill out all the items.

Graduate School of Clinical Dentistry, Ewha Womans University, 
Da-Hye Choi (Tel: 010-3088-9822)	
¢À Please read each of the following questions and mark it as 'O' or ¡®¡î¡¯.
1. What is your gender?
(1) Male  (2) Female
2. How old are you?
(1) 10-19 years  (2) 20-29 years  (3) 30-39 years  (4) 40-49 years (5) 50-59years (6) 60 and older
3. What is your occupation?
(1) Student (2) Office worker (3) Production worker (4) healthcare worker (5) Other (       )
4.Have you ever had or are suffering from the following diseases?
                                       (check if applicable.)
¡¤ cardiac disease                (           ) 
¡¤ cardiac pacemaker       (           ) 
¡¤ high blood pressure                (           ) 
¡¤ stroke             (           ) 
¡¤ rheumatic fever            (           ) 
¡¤ kidney disease             (           ) 
¡¤ hepatitis or liver disease       (           ) 
¡¤ diabetes                  (           ) 
¡¤ tuberculosis                  (           ) 
¡¤ STD                  (           ) 
¡¤ anemia                  (           ) 
¡¤ hemophilia                (           ) 

5. Do you smoke? (Yes, No)
Average number of cigarettes smoked per day:       cigarettes

6. Have you recently received a dental treatment?
(1) within a month (2) within 1-6 months (3) within 6-12 months (4) over 1 year 

7. Do you have your teeth scaled at the dentist regularly?
(1) Yes.(Answer 7-1.)
(2) No.

7-1 If so, how often do you get it?
(1) 1-3 months (2) 4-6 months (3) 6-12 months (4) 1-2 years
(5) over 2 years

8. How many times do you brush a day?
(1) once/day   (2) twice/day  (3) 3 times/day (4) more than 4 times/day

9. Please check all of your brushing times during the day.
(1) after each meal (    )    (2) before breakfast (    )  (3) after breakfast (    ) (4) after lunch (    ) (5) after dinner (    ) 

10.How often do you change toothbrush? (      ) months

11. Do you use oral care products (e.g., dental floss, tongue cleaner, mouthwash) other than toothbrush?
(1) Yes (Answer 11-1)  (2) No

11-1. If you use oral hygiene products (except toothpicks), what do you use?
(1) floss    (2) interdental brush   (3) mouthwash (Listerine, etc.) 
(4) tongue cleaner (5) waterpik (6) others (               ) 


 Thank you for your answer.
